# Surface Modification of Titanium Using Nano-Hydroxyapatite: A Comparative Microhardness Study

**DOI:** 10.7759/cureus.108531

**Published:** 2026-05-08

**Authors:** Shazia Kosar, Mohd Ali, Vidhi Sangra, Naveen Ambata, Priyanka Vhanmane, Parneet Kaur

**Affiliations:** 1 Department of Prosthodontics and Crown and Bridge, Government Dental College and Hospital, Srinagar, IND; 2 Department of Oral and Maxillofacial Surgery, Ivory Harbor The Dental Clinic, Jammu, IND; 3 Department of Prosthodontics and Crown and Bridge, Vishnu Dental College, Bhimavaram, IND; 4 Department of Periodontology, Bharati Vidyapeeth (Deemed to be University) Dental College and Hospital, Sangli, IND; 5 Department of Oral and Maxillofacial Surgery, Desh Bhagat Dental College & Hospital, Mandi Gobindgarh, IND

**Keywords:** biomaterials, dental implants, nano-hydroxyapatite, surface microhardness, titanium

## Abstract

Background and aim

Surface modification of titanium implants is essential to enhance their mechanical and biological performance. Nano-hydroxyapatite has emerged as a promising biomimetic material capable of improving surface characteristics and potentially optimizing implant success. The aim of this study was to evaluate the effect of nano-hydroxyapatite on the surface microhardness of titanium discs.

Methods

This in vitro experimental study included 30 commercially pure titanium discs allocated into control (artificial saliva) and experimental (nano-hydroxyapatite) groups (n = 15 each). Baseline surface microhardness was measured using a Vickers microhardness tester (Shimadzu Corporation, Kyoto, Japan). The experimental group received nano-hydroxyapatite twice daily for 14 days, while the control group was maintained in artificial saliva. Posttreatment microhardness was assessed, and changes in the Vickers hardness number (VHN) were calculated. Data were analyzed using independent and paired t-tests, with significance set at p < 0.05.

Results

Baseline microhardness values were comparable between the control (224.57 ± 6.76 VHN) and experimental groups (223.13 ± 7.32 VHN) (p = 0.582). Posttreatment, the experimental group showed a significant increase in microhardness (241.65 ± 7.34 VHN), whereas the control group demonstrated a slight decrease (222.01 ± 7.53 VHN). The mean change in VHN was +18.52 ± 3.54 in the experimental group and -2.55 ± 2.73 in the control group, with a highly significant intergroup difference (p < 0.001).

Conclusions

Within the limitations of this study, nano-hydroxyapatite surface modification significantly improved the microhardness of titanium. This suggests enhanced surface durability and potential for better clinical performance. Clinically, such modifications may improve resistance to wear and deformation under load-bearing conditions. Additionally, improved surface characteristics may promote superior osseointegration, contributing to the long-term success of dental implants.

## Introduction

Titanium and its alloys are widely used in dentistry and implantology owing to their excellent mechanical properties, corrosion resistance, and biocompatibility. The success of titanium-based implants largely depends on their ability to achieve stable osseointegration, which is influenced by the surface characteristics of the material [1,2]. Surface properties such as roughness, chemical composition, and hardness play a critical role in determining cellular responses, protein adsorption, and overall implant integration. Despite the favorable properties of titanium, its bioinert nature may limit direct biological interactions, thereby necessitating surface modification strategies to enhance its bioactivity [3].

In recent years, surface modifications using bioactive materials have gained considerable attention [3]. Commonly used titanium surface modification methods include mechanical roughening, acid etching, bioactive coatings (such as hydroxyapatite), anodization, and laser-based techniques. Among these, nano-hydroxyapatite has emerged as a promising candidate owing to its chemical similarity to the mineral components of bone [4]. The biological apatite in human bone consists of approximately 60-70% inorganic mineral content, primarily in the form of hydroxyapatite with a calcium-to-phosphorus ratio of approximately 1.67, which closely matches that of synthetic nano-hydroxyapatite. Nano-hydroxyapatite exhibits superior surface reactivity, increased surface area, and enhanced biological affinity compared with its conventional counterparts [4]. These properties enable improved interaction with surrounding tissues and may contribute to better mechanical and biological performance of implant surfaces [5]. The application of nano-hydroxyapatite onto titanium surfaces is therefore considered a potential approach to improve surface characteristics, including microhardness, which is an important parameter reflecting resistance to surface deformation and wear [6].

Microhardness evaluation provides insights into the structural integrity and durability of implant materials under functional conditions [7]. Modifications that enhance surface hardness may contribute to improved longevity and clinical success of implants. However, there is limited literature evaluating the direct effect of nano-hydroxyapatite application on the microhardness of titanium surfaces under simulated oral conditions [8]. Hence, further investigations are required to establish its effectiveness and potential clinical relevance. This study aimed to evaluate the effect of nano-hydroxyapatite on the surface microhardness of titanium discs. The objectives were to (1) measure the baseline microhardness of titanium specimens; (2) assess changes in microhardness following nano-hydroxyapatite application; and (3) compare the results with a control group stored in artificial saliva to determine the efficacy of surface modification.

## Materials and methods

This experimental, laboratory-based study was conducted in the Department of Prosthodontics and Crown and Bridge, Government Dental College and Hospital, Srinagar, India, in collaboration with the Central Research Laboratory of the institution over a period of four months from January 2025 to April 2025. Because the study did not involve human participants, animal subjects, or biological tissues, ethical approval was not required.

The sample size was calculated a priori using G*Power software (version 3.1, Heinrich Heine University, Düsseldorf, Germany) for an independent sample t-test, assuming a large effect size (Cohen’s d = 0.8), α = 0.05, and power (1-β) = 0.80, yielding a minimum of 13 specimens per group. To compensate for potential specimen loss, 15 samples were included in each group (n = 30).

Commercially pure titanium discs (Grade IV), measuring 10 mm in diameter and 2 mm in thickness, were used as study specimens. Nano-hydroxyapatite powder (Sigma-Aldrich, St. Louis, Missouri, USA) was selected as the test material because of its biomimetic properties and established remineralization potential. Nano-hydroxyapatite powder (Sigma-Aldrich), with a particle size ranging from approximately 20-80 nm and purity ≥97%, was used. The material was selected due to its nanoscale morphology and compositional similarity to biological apatite. Artificial saliva was prepared using a standardized formulation to simulate the oral environment. All materials were handled according to the manufacturer’s instructions to ensure consistency and reproducibility.

Thirty titanium discs were prepared and standardized prior to the experiment. The surfaces were sequentially polished using 400, 600, 800, and 1200 grit silicon carbide abrasive papers under continuous water irrigation to achieve a uniform, smooth finish. Final polishing was performed using an alumina slurry to obtain a mirror-like surface. The specimens were then ultrasonically cleaned in distilled water for 10 minutes to remove any residual debris and dried at room temperature. Baseline surface microhardness was measured using a Vickers microhardness tester (Shimadzu Corporation, Kyoto, Japan) with a load of 200 g applied for 15 seconds. Three indentations were made on each specimen at different locations, and the mean values were recorded.

The prepared specimens were allocated into two groups, with 15 discs in each group. The control group specimens were immersed in artificial saliva for the entire duration of the study, without any surface treatment. The specimens in the experimental group were treated with a nano-hydroxyapatite suspension.

A nano-hydroxyapatite suspension was prepared by dispersing the powder in distilled water at a concentration of 10% (w/v), followed by continuous stirring using a magnetic stirrer to ensure a homogeneous mixture. For the experimental group, the prepared suspension (approximately 0.5 mL) was applied uniformly onto the surface of each titanium disc using a microbrush. The material was allowed to remain in contact with the surface for five minutes to facilitate interaction. Following application, the discs were gently rinsed with distilled water to remove excess material. The application procedure was performed twice daily for 14 days. All specimens from both groups were stored in artificial saliva at 37 °C in an incubator to simulate intraoral conditions. Artificial saliva (Wet Mouth^®^, ICPA Health Products Ltd, Mumbai, India) was used as the storage medium. The formulation consisted of sodium carboxymethylcellulose, potassium chloride (0.4 g/L), sodium chloride (0.4 g/L), calcium chloride dihydrate (0.795 g/L), magnesium chloride hexahydrate (0.005 g/L), potassium dihydrogen phosphate (0.69 g/L), and sodium fluoride (0.001 g/L), adjusted to a pH of approximately 6.8-7.0 to simulate the oral environment.

**Figure 1:**
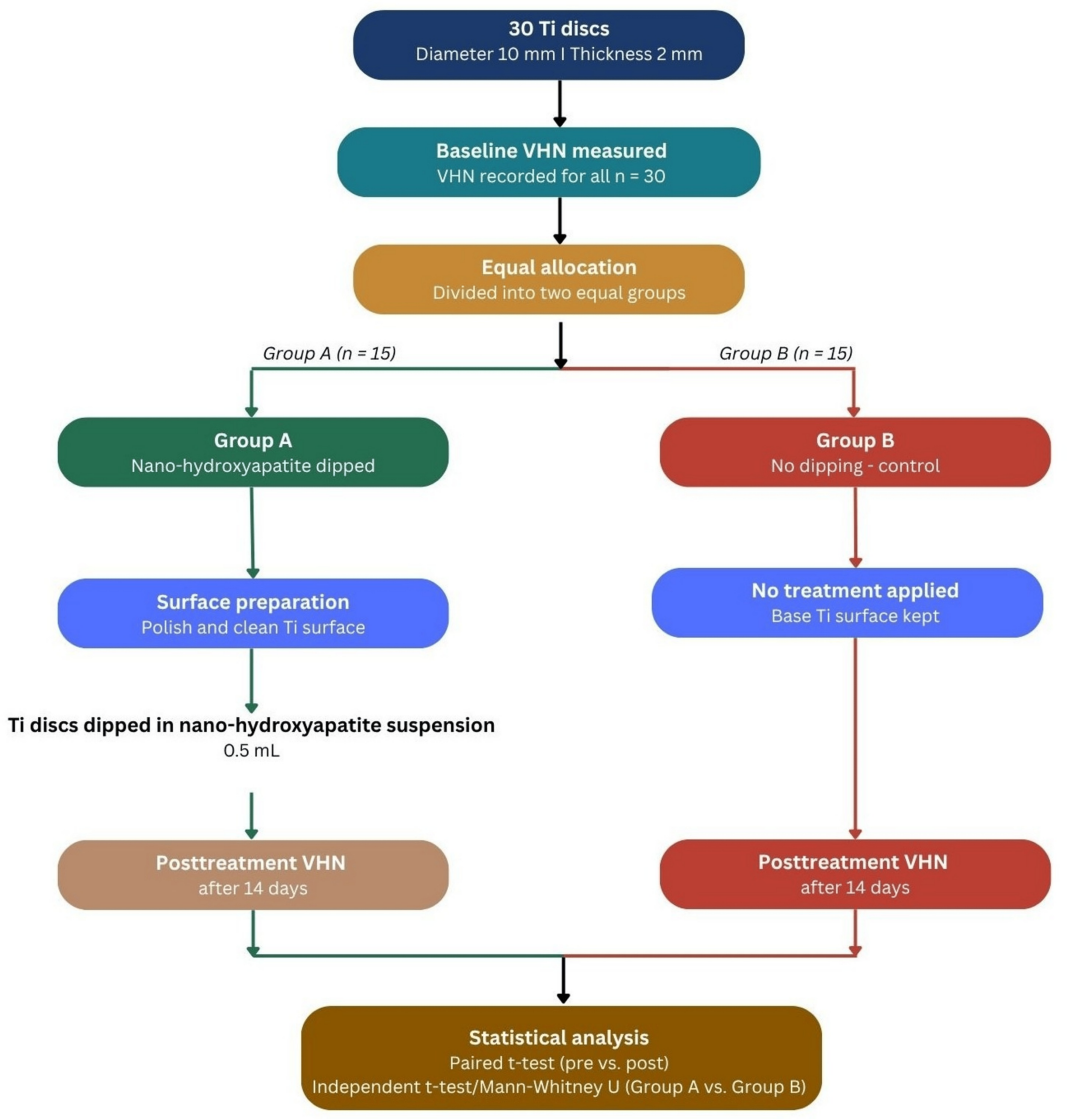
Study flowchart HA, nano-hydroxyapatite; post, posttreatment; pre, pretreatment; Ti, titanium; VHN, Vickers hardness number This figure was created using Canva (Canva Pty Ltd., Sydney, Australia).

At the end of the experimental period, all specimens were rinsed with distilled water and dried. Surface microhardness was reassessed using the same Vickers microhardness test parameters as those used for baseline measurements. Three indentations were made on each specimen, and the mean value was calculated to obtain the final microhardness value. The study flowchart is presented in Figure 1.

The primary outcome measure was the change in surface microhardness, expressed as the Vickers hardness number (VHN), before and after treatment. Differences between the baseline and posttreatment values were calculated for each specimen. Statistical analysis was performed using IBM SPSS Statistics for Windows, version 26.0 (released 2018; IBM Corp., Armonk, NY, USA). Data are expressed as mean ± SD. Normality of distribution was assessed using the Shapiro-Wilk test, and homogeneity of variance was evaluated using Levene’s test. Parametric tests were applied when the data followed a normal distribution. Intergroup comparisons (control vs. experimental) at baseline, posttreatment, and in changes in VHN were performed using the independent samples t-test. Intragroup comparisons (baseline vs. posttreatment) were performed using a paired t-test. All statistical tests were two-tailed, with a significance level set at p < 0.05 and a 95% CI.

## Results

At baseline, the control and experimental groups demonstrated comparable surface microhardness values. The mean VHN in the control group was 224.57 ± 6.76, while the experimental group showed a mean value of 223.13 ± 7.32. Intergroup comparisons using the independent samples t-test revealed no statistically significant difference between the groups (t = 0.557; p = 0.582), confirming baseline homogeneity of the specimens. Intergroup comparison at the posttreatment stage revealed a statistically significant difference in surface microhardness between the two groups. The experimental group demonstrated a significantly higher mean VHN (241.65 ± 7.34) than the control group (222.01 ± 7.53) (p < 0.001). Furthermore, the mean change in microhardness (ΔVHN) was markedly higher in the experimental group (+18.52 ± 3.54) than in the control group (-2.55 ± 2.73), with the difference being highly significant (p < 0.001) (Table 1).

**Table 1:** Intergroup comparison of surface microhardness values between control and experimental groups Data are presented as mean ± SD. ^***^ p < 0.001 indicates a highly statistically significant difference. Δ = change from baseline to posttreatment; independent samples t-test applied; degrees of freedom = 28 Control group: titanium discs immersed in artificial saliva without surface treatment; experimental group: titanium discs treated with nano-hydroxyapatite suspension VHN, Vickers hardness number

Comparison	Control group (mean ± SD)	Experimental group (mean ± SD)	t-value	p-value
Baseline VHN	224.57 ± 6.76	223.13 ± 7.32	0.557	0.582
Posttreatment VHN	222.01 ± 7.53	241.65 ± 7.34	-7.233	<0.001^***^
Change in VHN (Δ)	-2.55 ± 2.73	+18.52 ± 3.54	-18.275	<0.001^***^

Intragroup analysis demonstrated distinct trends within each group during the 14-day experimental period (Table 2). In the control group, a statistically significant reduction in microhardness was observed, with mean VHN decreasing from 224.57 ± 6.76 at baseline to 222.01 ± 7.53 posttreatment (mean difference: -2.55 ± 2.73; p = 0.003). In contrast, the experimental group exhibited a highly significant increase in surface microhardness, with mean VHN rising from 223.13 ± 7.32 at baseline to 241.65 ± 7.34 after treatment (mean difference: +18.52 ± 3.54; p < 0.001). Overall, the results demonstrated that nano-hydroxyapatite application significantly enhanced the surface microhardness of titanium discs compared with the control condition. Although artificial saliva alone resulted in a slight but statistically significant reduction in microhardness, nano-hydroxyapatite treatment produced a substantial increase over the 14-day period.

**Table 2:** Intragroup comparison of baseline and posttreatment microhardness values (VHN) Data are presented as mean ± SD. ^*^ p < 0.05 indicates a statistically significant difference. ^***^ p < 0.001 indicates a highly statistically significant difference. Paired t-test applied; degrees of freedom = 14 for each group Control group: titanium discs immersed in artificial saliva without surface treatment; experimental group: titanium discs treated with nano-hydroxyapatite suspension VHN, Vickers hardness number

Group	N	Baseline (mean ± SD)	Posttreatment (mean ± SD)	t-value	p-value
Control group (artificial saliva)	15	224.57 ± 6.76	222.01 ± 7.53	3.628	0.003^*^
Experimental group (nano-hydroxyapatite)	15	223.13 ± 7.32	241.65 ± 7.34	-20.277	<0.001^***^

## Discussion

This study evaluated the effect of nano-hydroxyapatite on the surface microhardness of titanium discs under simulated oral conditions. The findings demonstrated a statistically significant increase in VHN in the experimental group following nano-hydroxyapatite application, whereas the control group exhibited a slight but significant reduction in microhardness after storage in artificial saliva. These results suggest that nano-hydroxyapatite plays a beneficial role in enhancing the surface properties of titanium, which may be critical for improving implant performance.

The significant improvement in microhardness observed in the experimental group can be attributed to the biomimetic and bioactive nature of nano-hydroxyapatite. Owing to its chemical similarity to the mineral phase of bone, nano-hydroxyapatite can form a stable interface with titanium surfaces. Its nanoscale size provides a higher surface area and increased reactivity, facilitating better adhesion and deposition onto the titanium substrate. This likely resulted in the formation of a reinforced surface layer, thereby increasing resistance to indentation and surface deformation. Similar findings were reported by Surmenev et al. [9], who demonstrated that calcium phosphate coatings significantly improve bone regeneration.

The present findings are consistent with those of the in vivo study by Oliveira et al. [10], which demonstrated that nanometric hydroxyapatite-coated implant surfaces exhibited improved nanomechanical properties and enhanced bone response, even under compromised systemic conditions such as diabetes. Similarly, Lin et al. [11] reported that titanium implants modified with hydroxyapatite nanoparticles significantly improved early bone implant fixation, highlighting the positive influence of nanoscale surface modifications on both mechanical stability and biological integration. A previous review has reported that nano-hydroxyapatite-coated implant surfaces significantly enhanced osteogenic gene expression and promoted improved osseointegration, thereby corroborating the positive effect of nano-hydroxyapatite on enhancing the functional and biological properties of titanium surfaces [8].

The control group showed a minor but statistically significant reduction in microhardness after immersion in artificial saliva. This finding may be explained by potential erosive or ionic interactions of the storage medium with the titanium surface over time. Although artificial saliva is used to simulate oral conditions, prolonged exposure may lead to subtle surface alterations, including ion exchange or microstructural changes, which can slightly reduce hardness. Similar trends have been noted in studies evaluating the effect of oral environments on dental materials, where prolonged immersion resulted in marginal degradation of surface properties [12].

The findings of the present study are further supported by several in vivo investigations demonstrating the beneficial effects of nano-hydroxyapatite surface modification on titanium implants. Júnior et al. [13] reported that nano-hydroxyapatite-coated implant surfaces, particularly when combined with leukocyte-platelet-rich fibrin, significantly enhanced bone formation and implant stability in rat models, indicating improved biological performance. Similarly, Bergamo et al. [14] demonstrated that physicochemical surface modifications, including hydroxyapatite incorporation, synergistically improve osseointegration when combined with optimized implant macrogeometry. Furthermore, Almeida et al. [15] showed that nanostructured hydroxyapatite coatings significantly enhance osseointegration in low-density bone conditions. Rathi et al. [16] reported significantly enhanced surface microhardness, supporting the reinforcing effect observed in the present study. These findings collectively corroborate the results of the present study, suggesting that nano-hydroxyapatite not only improves the biological integration of titanium implants but also contributes to enhanced surface characteristics, including microhardness.

From a clinical perspective, the enhancement of titanium surface microhardness has important implications. Increased surface hardness can improve wear resistance and durability of dental implants, particularly in load-bearing regions. Moreover, surface modification with nano-hydroxyapatite may promote osseointegration by providing a more favorable surface for bone cell attachment and proliferation. This dual benefit of mechanical reinforcement and biological enhancement suggests that nano-hydroxyapatite is a promising adjunct for implant surface engineering.

This study has several limitations that should be considered when interpreting the findings. As an in vitro investigation, the experimental conditions do not fully replicate the complex biological, chemical, and mechanical environment of the oral cavity, including salivary flow, pH variations, microbial activity, and functional loading, and therefore, the results cannot be directly extrapolated to clinical situations. The study evaluated only surface microhardness, which reflects resistance to surface deformation but does not provide a comprehensive assessment of implant surface performance, as parameters such as surface roughness, wettability, adhesion strength, and wear resistance were not assessed. In addition, no surface characterization techniques, such as scanning electron microscopy or compositional analyses, were performed to confirm the deposition of nano-hydroxyapatite or to elucidate the mechanism underlying the observed changes in microhardness. The concentration of artificial saliva was not periodically measured, although a standardized formulation was used and refreshed daily to maintain consistency. Furthermore, blinding during outcome assessment was not implemented, which may introduce a potential risk of measurement bias despite the use of standardized instrument-based testing methods. Future studies incorporating in vivo conditions, comprehensive surface analyses, and enhanced methodological controls are recommended to validate these findings.

## Conclusions

Within the limitations of this in vitro study, nano-hydroxyapatite application resulted in a significant increase in the surface microhardness of titanium discs compared to untreated controls. The observed improvement in VHN suggests enhanced resistance to surface deformation under controlled laboratory conditions, while artificial saliva alone produced a slight reduction in hardness. These findings indicate that nano-hydroxyapatite is capable of modifying titanium surface properties in vitro. However, extrapolation of these results to clinical performance should be approached with caution, as the complex biological and functional conditions of the oral environment were not replicated. Further in vivo and long-term studies are necessary to determine clinical relevance.
